# High Diagnostic Yield of Targeted Next-Generation Sequencing in a Cohort of Patients With Congenital Hypothyroidism Due to Dyshormonogenesis

**DOI:** 10.3389/fendo.2020.545339

**Published:** 2021-02-22

**Authors:** Athanasia Stoupa, Ghada Al Hage Chehade, Rim Chaabane, Dulanjalee Kariyawasam, Gabor Szinnai, Sylvain Hanein, Christine Bole-Feysot, Cécile Fourrage, Patrick Nitschke, Caroline Thalassinos, Graziella Pinto, Mouna Mnif, Sabine Baron, Marc De Kerdanet, Rachel Reynaud, Pascal Barat, Mongia Hachicha, Neila Belguith, Michel Polak, Aurore Carré

**Affiliations:** ^1^ INSERM U1016, Cochin Institute, Faculté de Médecine, Université Paris Descartes, Sorbonne Paris Cité, Paris, France; ^2^ IMAGINE Institute affiliate, Paris, France; ^3^ Pediatric Endocrinology, Gynecology and Diabetology Unit, Hôpital Universitaire Necker-Enfants Malades, AP-HP, Paris, France; ^4^ Laboratory of Human Molecular Genetics, Medicine School, University of Sfax, Sfax, Tunisia; ^5^ Pediatric Immunology, Department of Biomedicine, University of Basel, Basel, Switzerland; ^6^ Pediatric Endocrinology, University Children’s Hospital Basel, University of Basel, Basel, Switzerland; ^7^ INSERM U1163, IMAGINE Institute, Translational Genetics, Université Paris Descartes, Sorbonne Paris Cité, Paris, France; ^8^ Genomics Platform, INSERM UMR 1163, Paris Descartes Sorbonne Paris Cite University, Imagine Institute, Paris, France; ^9^ Bioinformatics Platform, Paris Descartes University, IMAGINE Institute, Paris, France; ^10^ Endocrinology Department, CHU Hedi Chaker, Sfax, Tunisia; ^11^ Pediatrics Department, CHU Nantes, Nantes, France; ^12^ Pediatrics Department, CHU Rennes, Rennes, France; ^13^ Pediatrics Department, CHU La Timone, Marseille, France; ^14^ CHU de Bordeaux, Pediatric Endocrinology, Bordeaux, France; ^15^ Pediatrics Department, CHU Hedi Chaker, Sfax, Tunisia; ^16^ Medical Genetics Department, CHU Hedi Chaker, Sfax, Tunisia; ^17^ Centre de Référence des Maladies Endocriniennes Rares de la Croissance et du Développement, Necker-Enfants Malades University Hospital, Paris, France; ^18^ Centre Régional de Dépistage Néonatal (CRDN) Ile de France, Paris, France

**Keywords:** congenital hypothyroidism, dyshormonogenesis, mutations, targeted next-generation sequencing, gland *in situ*

## Abstract

**Objective:**

To elucidate the molecular cause in a well-characterized cohort of patients with Congenital Hypothyroidism (CH) and Dyshormonogenesis (DH) by using targeted next-generation sequencing (TNGS).

**Study design:**

We studied 19 well-characterized patients diagnosed with CH and DH by targeted NGS including genes involved in thyroid hormone production. The pathogenicity of novel mutations was assessed based on *in silico* prediction tool results, functional studies when possible, variant location in important protein domains, and a review of the recent literature.

**Results:**

TNGS with variant prioritization and detailed assessment identified likely disease-causing mutations in 10 patients (53%). Monogenic defects most often involved *TG*, followed by *DUOXA2*, *DUOX2*, and *NIS* and were usually homozygous or compound heterozygous. Our review shows the importance of the detailed phenotypic description of patients and accurate analysis of variants to provide a molecular diagnosis.

**Conclusions:**

In a clinically well-characterized cohort, TNGS had a diagnostic yield of 53%, in accordance with previous studies using a similar strategy. *TG* mutations were the most common genetic defect. TNGS identified gene mutations causing DH, thereby providing a rapid and cost-effective genetic diagnosis in patients with CH due to DH.

## Introduction

Congenital hypothyroidism (CH) is the most common neonatal endocrine disorder, with an incidence of 1/2,500–3,500 newborns (1, 2). Among patients with CH, 65% have thyroid dysgenesis (TD), with a large phenotypic spectrum encompassing athyreosis, thyroid ectopy, hypoplasia of an orthotopic gland, and hemithyroid (3). In the remaining 35% of patients, CH is due to dyshormonogenesis (DH) with a thyroid gland *in situ* (GIS) with or without goiter. DH may lead to goiter formation due to thyroid tissue overstimulation by increased TSH levels. Most cases of DH are due to mutations in *TG, TPO, SLC5A5/NIS, SLC26A4/PDS*, *IYD/DEHAL1*, *DUOX2*, *DUOXA2, DUOX1, DUOXA1*, and *SLC26A7*, which are involved in thyroid hormone production and usually inherited on an autosomal recessive basis (4–6).

The proportion of patients with CH due to DH who receive a molecular diagnosis varies widely across studies, from 20 to 60% (7–13). Factors contributing to this variability include differences in patient phenotypes, clinical characterization of the patients (imaging techniques, as thyroid ultrasound and scintigraphy, perchlorate test, thyroglobulin measurement), geographic origin, and mainly variant classification.

The objective of this study was to assess the diagnostic yield of targeted Next Generation Sequencing (TNGS) in a cohort of 19 well-characterized patients with CH due to DH. We also report the results of an extensive literature review of studies describing genetic findings in patients with CH, with special attention to those having DH.

## Materials and Methods

### Patients

Nineteen patients with permanent primary CH, referred by various centers in France, Tunisia (n = 2), and the United States of America (USA) (n = 1) were included in the study. Diagnosis of primary CH was based on systematic newborn screening in France and increased venous TSH at control blood sample. Cut-off in blood spot for newborn screening is 15 mIU/L in France and 20 mIU/L in New York (USA); no newborn screening is available in Tunisia. CH was diagnosed at birth for all patients except the patient from USA and the Tunisian patients diagnosed during the first months or year of life, respectively, due to clinical symptoms suggesting hypothyroidism and confirmed by high TSH levels and low free T4 levels (FT4), according to laboratory reference values. Inclusion criteria of 19 patients were CH with GIS and at least one of the following: clinical goiter (≥2SD), available thyroid scintigraphy providing an evaluation of thyroid position and radionuclide uptake, and a perchlorate discharge test performed. This study was approved by French Biomedecine Agency. Written, informed consent was obtained from the individuals and minors’ legal guardian for the publication of any potentially identifiable images or data included in this article.

### Detection of Mutations

We designed a TNGS panel (HypothySeq NGS) of 78 genes including genes known to be associated with CH (thyroid dysgenesis; dyshormonogenesis; thyroid hormone transport protein defects; and inborn errors in thyroid hormone membrane transport, metabolism, or action) and candidate genes that have been identified in animal models (mouse and zebrafish knock-out models) or by microarray assays but not yet validated in humans. Sensitivity (false-negative rate) of the panel was assessed in positive controls with known thyroid disease-causing mutations, including mutations in dyshormonogenesis genes, and specificity (false-positive rate) in healthy individuals previously screened using whole exome sequencing for another research project.

TNGS on HiSeq 2500 system (Illumina, San Diego, CA) and bioinformatics analyses were already described (14). Mean coverage for each gene is reported **Table 1**.

**Table 1:** Reference transcript and average coverage of genes responsible for CH and DH.

	Reference transcript	Average coverage
TG	NM_003235	614
TPO	NM_000547	694
DUOX2	NM_014080	586
DUOXA2	NM_207581	516
NIS/SLC5A5	NM_000453	577
PDS/SLC26A4	NM_000441	720
DEHAL1/IYD	NM_001164694, NM_203395	580

### Prioritizing Strategy for Filtering Pathogenic Variants

Variants (including frameshift mutations, missense and nonsense mutations, and splicing-site mutations) identified in known dyshormonogenesis genes were considered for the analysis. If available, functional data and segregation analysis results were taken into consideration.

Inheritance is recessive for *TPO*, *TG*, *DUOX2*, *DUOXA2*, *SLC5A5*, and *SLC26A4* and dominant for *PAX8*. Genome variations were defined using PolyDiag in-house software for TNGS, which filters out irrelevant and common polymorphisms based on frequencies extracted from the following public databases: US National Center for Biotechnology Information database of single nucleotide polymorphisms (SNP) (dbSNP), 1000 Genomes, Exome Variant Server and Exome Aggregation Consortium (ExAC). Consequences of mutations on protein function were predicted using three algorithms: Polyphen2, SIFT, and Mutation Taster. Mutations were ranked based on the impact of each variant predicted by combined annotation-dependent depletion (CADD) then compared using the mutation significance cut-off, which is a gene-level specific cutoff for CADD scores.

For deletions or insertions in exons, information on the variants was sought in similar published studies. Sanger sequencing was performed to validate and segregate the identified variants (3500xL Genetic Analyzer, Thermo Fisher Scientific, Waltham, MA). Primer sequences are shown in **Table 2**.

**Table 2:** Primers sequences to verify variants by PCR and Sanger sequencing.

Gene	Exon	Primer Forward	Primer Reverse
PAX8	3	GGCTCTGGCTAAATCCCTGTCTAA	TCCCTGCCTGATTGTTCAGCAT
PAX8	7	TGCAGGAAGGTCGGCTTGTT	GACAGCCAGCCAAGCTCTTCA
SLC5A5	9_10	GATGGTGTGGACGGTCTCTC	TAATGGGAAAGAGGGAAAGG
TG	5	GAGTGCATATGCTGCTCGAC	TCAAGGTGAGTGTGGGCTG
TG	6	TTCCTTTTCACTAGGCGTGG	GCAGGCAGTCACTCTAGCTG
TG	7	AACTTTGAAACCCAAGAGGC	AGGTCAGGGCTTCCTTTCTG
TG	9	CTCTGTGCCAGAAGATGTGG	CTGTACTGCATTGGGTCAGG
TG	22	TAGGAGTCAGGGGATTCCAG	AGCCCTTGAGACTACTCCCC
TG	26	TCCAACTCTGCCATGTTTTG	CAGCTCCATGTTGTGTGTCC

## Results

### Clinical Description of the Study Cohort

We studied 19 index cases of CH due to dyshormonogenesis **(** **Table 3** **)**. Among them, 16 were born in France [two of them (patients #4 and #18) born in France to consanguineous parents, and of Moroccan and Turkish origin, respectively] and were diagnosed with primary CH at birth by routine neonatal screening (TSH cutoff, 15 mIU/L) followed by a venous-blood TSH assay. CH was diagnosed at day 75 of age in one patient (#19) born in the USA and at 2 months and 5 months of age in two patients (#14 and #15 respectively) born in Tunisia. These three patients underwent evaluation for clinical symptoms suggesting hypothyroidism and were found to have high TSH levels and low FT4 levels. Of the 19 patients, eight had a family history of CH and five were born to consanguineous parents, including one with an affected sibling (#1). A goiter was evident at diagnosis in nine patients including two (#3 and #16) diagnosed with goiter in utero. Fetal goiter was diagnosed during one of the routine fetal sonograms performed in France during pregnancy at 13, 22, and 32 gestational weeks. In our cases, the diagnosis was made on the second ultrasound, around 22 gestational week and treated with intraamniotic levothyroxine injections, mean dose between 200 and 400 micrograms/injection. The response to levothyroxine treatment was assessed by decrease in thyroid size in fetal ultrasound and/or normalization of fetal thyroid hormones. According to the European Society for Paediatric Endocrinology guidelines (15) the FT4 level at diagnosis indicated severe CH in nine patients (FT4<5 pmol/L) and moderate CH in 1 patient (FT4, 10–15 pmol/L). Thyroglobulin was not assayed routinely. A perchlorate discharge test was performed in 14 patients, of whom 11 had a positive result >10%.

**Table 3:** Clinical description of cohort of patients with CH due to DH.

Patient	Country of origin	Consanguineous parents	Age / Sex	Age at diagnosis	TSH at diagnosis (mIU/L)	free T4 at	Thyroglobulin	Goiter at diagnosis	Cervical Ultrasound	Thyroid Scintigraphy	Perchlorate test	Associated features	Familial cases
1	France	No	19 y / M	D9	470	3.5	NA	No	NA	Normal position - elevated uptake	11%	–	CH in a brother with similar clinical features
2	France	No	30 y / M	D19	49	NA	1.5	Yes	Normal position - RL 46x25 mm - LL 45x30 mm	Normal position - elevated uptake	N	–	–
3	France	No	6 y / M	D19	143	8.4	NA	Yes. In utero	NA	Normal position, normal uptake	10.4%	–	Subclinical non-autommune hypothyroidism in mother and grand-mother
4	Morocco	Yes	9 y / F	D19	100	NA	2,512	Ye, mild	Normal position - RL 18x9 mm - LL 20x10 mm	Normal position - elevated uptake	84%	–	–
5	France	No	19 y / M	D14	657	3	NA	No	NA	Normal position - elevated uptake	50%	–	–
6	Tunisia	Yes	11 y / M	D4	637	undetectable	NA	Yes	Normal position	Normal position, normal uptake	N	–	–
7	France	No	6 y / F	D8	443	6.1	1.2	No	Normal position - RL 21x10 mm - LL 17x10 mm	Normal position	13%	–	–
8	France	No	24 y / F	D3	110	6.2	NA	No	Normal position	Normal position - elevated uptake	42%	–	–
9	France	No	22 y / M	D3	high (NA)	NA	NA	Yes, mild	Normal position	NA	NA	–	2 brothers with CH and similar clinical presentation
10	France	No	17 y / F	D13	580	< 1.8	NA	Yes	Normal position RL 30x6 mm - LL 30x3.5 mm	No Iodine uptake	–	–	–
11	France	No	3 y / M	D10	300	14	NA	No	NA	Normal position - normal uptake	50%	Unilateral cryptorchidism - no renal anomalies	CH and gland in situ (mother) - urinary tract duplication (father)
12	France	No	7 y / F	D9	53.8	NA	431	No	Normal position - length: RL 21 mm - LL 21 mm	Normal position - normal uptake	25%		–
13	France	No	8 y / F	D9	111.5	< 5	NA	No	Normal position - RL 7.7x5 mm - LL 8.1x5.9 mm	Normal position	57%	–	CH in the twin sister
14	Tunisia	Yes	32 y / M	2 M	90	2	35	No	Normal position	No Iodine uptake	NA	–	CH (brother. similar clinical presentation) - CH (uncle)
15	Tunisia	Yes	24 y / F	5 M	> 60	< 0.5	NA	No	Normal position	No Iodine uptake	NA	–	–
16	France	No	15 y / M	D1	180	4.9	NA	Ye, in utero	Normal position	Normal position	26%	–	–
17	France	No	26 y / M	D3	37.6	20	71	Yes	Normal position - RL 6x4x11 mm - LL 4x5x10 mm	Normal position - normal uptake	N	–	CH in the sister
18	Turkey	Yes	9 y / F	D3	591	3.5	2,110	Yes	Normal position - RL 40x20x10 mm - LL 40x19.5x11 mm	Normal position	27%	–	
19	United States	No	5 y / F	D75	755	1.2	NA	No	Normal position	Normal position - reduced uptake	NA	Prematurity. No intrauterine growth	CH in the twin sister
Normal values	Age	TSH (mIU/L)	T4 (pmol/L)							
	2 – 7 D	1.1 – 15.6	11.6 – 36.0							
	8– 15 D	0.87 – 7.8	9.5 – 28.9							
	16– 30 D	0.82 – 6.9	9.3 – 23.5							
	1 month – 1y	0.80- 6.05	8.3 – 18.6							
Normal values	TG	20-70ng/mL								

NA, not available; N, normal; D, days; M, months; y, years; M, male; F, female; RL, right lobe; LL, left lobe. Normal perchlorate discharge test is considered when the discharge is less than 10%.

### Genetic Results and Diagnostic Yield

**Figure 1:**
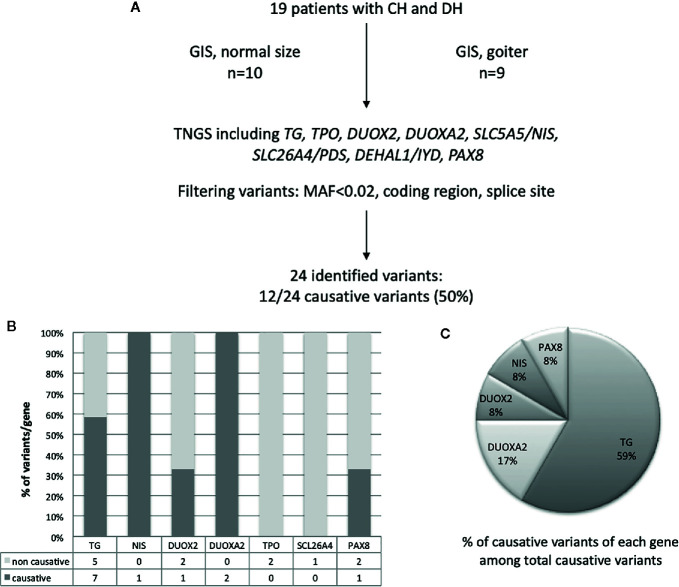
Schematic representation of found variants in the cohort of patients with CH due to DH**. (A)** Flowchart of the selection and distribution of variants identified by TNGS in 19 patients, including *TG*, *TPO*, *DUOX2*, *DUOXA2*, *SLC5A5*/*NIS*, *SLC26A4*, *DEHAL1*/*IYD*, and *PAX8* **(B)** Distribution of variants detected for each gene and absolute numbers of non-causative and causative variants. **(C)** Distribution of causative variants for each gene calculated relative to the total number of causative variants.

**Figure 2:**
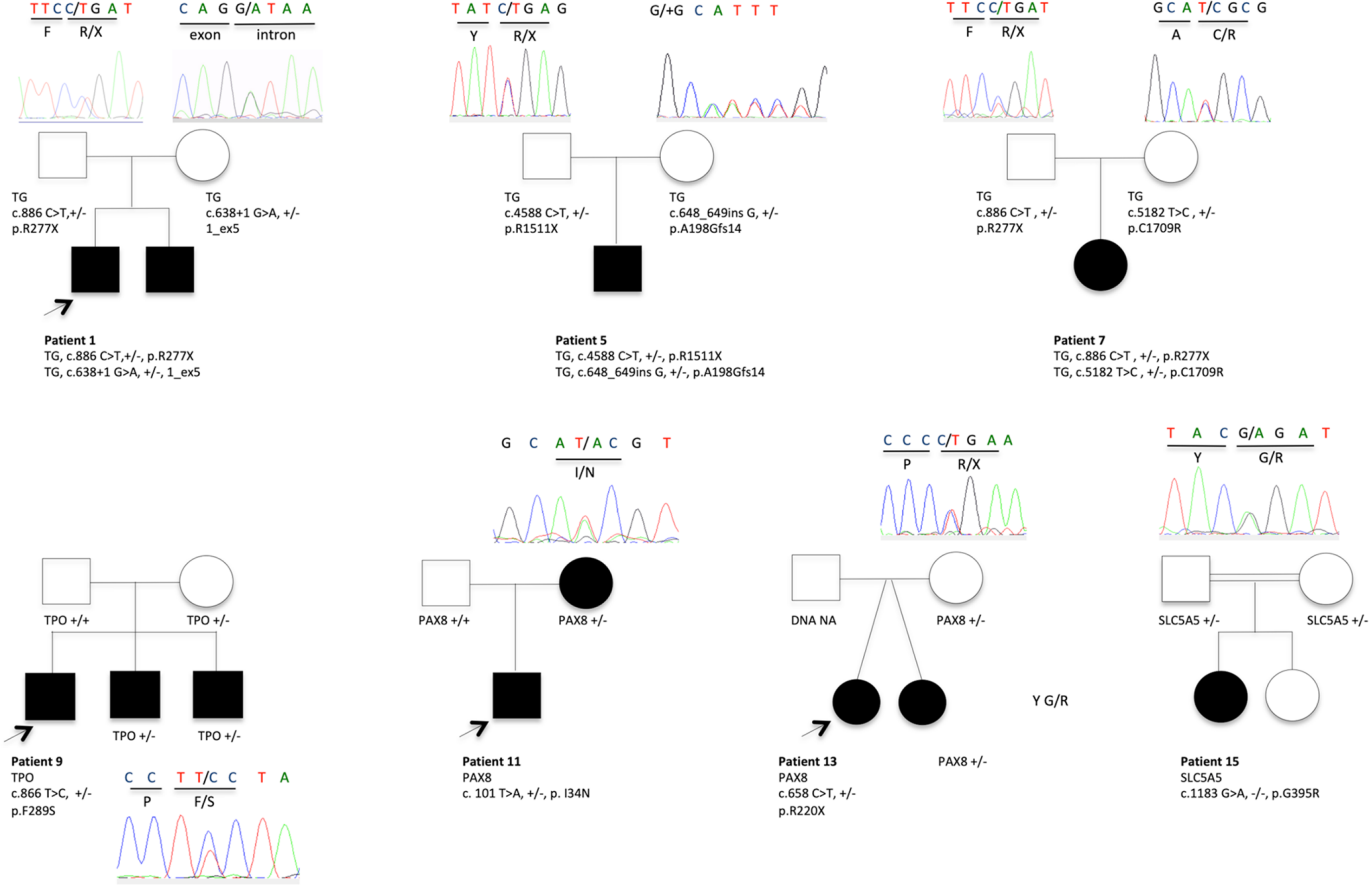
Pedigrees showing causative variant distribution and segregation in seven families. The patients with CH and DH are represented by filled boxes. In familial forms, the index patient is indicated by an arrow. Representative chromatograms are shown for each family member. NA, not available for DNA sampling.

**Figure 3:**
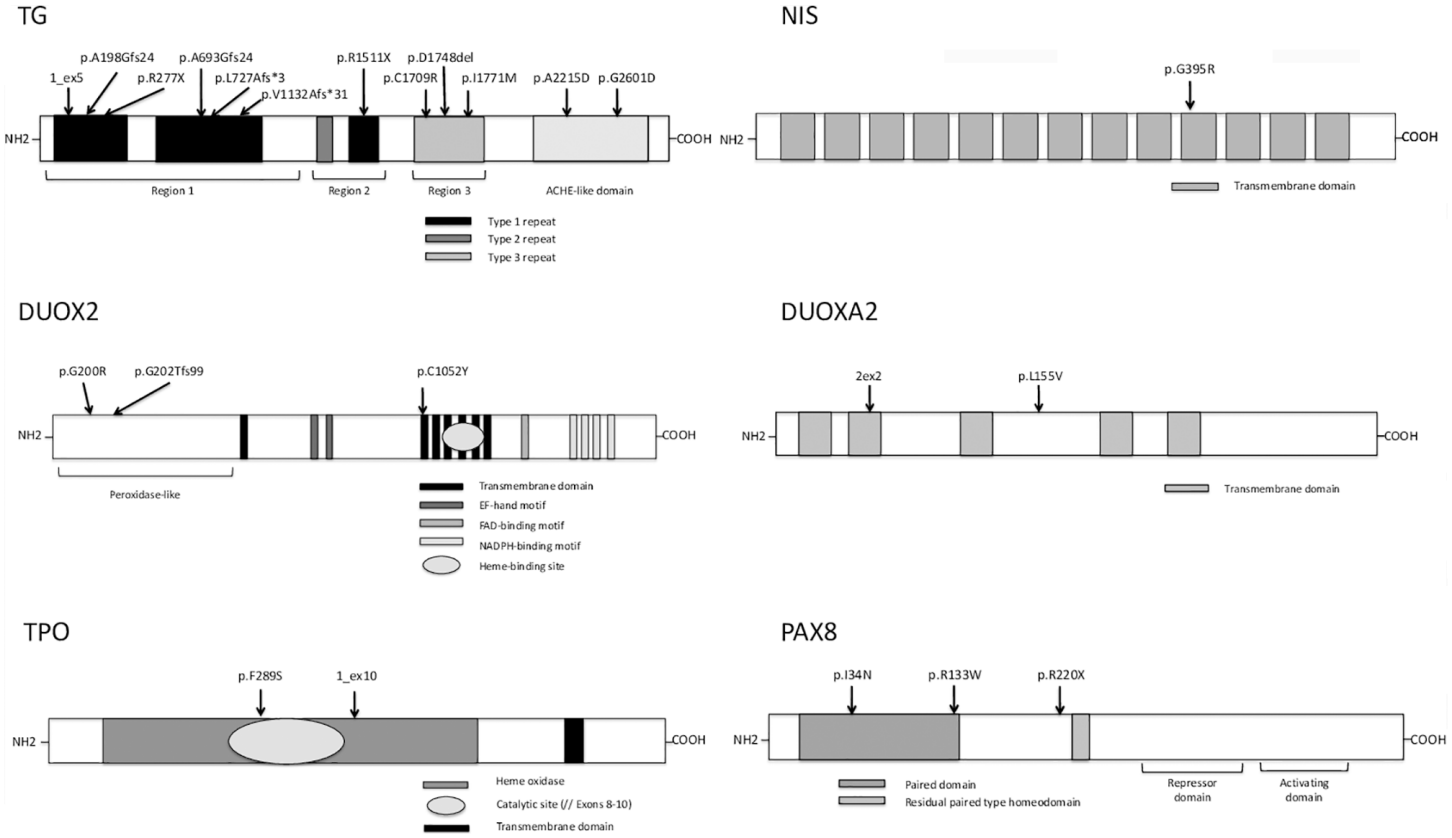
Variants identified in the present study with their location in the protein domains of thyroglobulin (TG), sodium-iodide symporter (NIS), dual oxidase 2 (DUOX2), dual oxidase maturation factor 2 (DUOXA2), thyroid peroxidase (TPO), and paired-box protein 8 (PAX8). TG: arrows show the positions of identified mutations in key structural domains including the repetitive type 1, type 2, and type 3 cysteine-rich regions (shown in black, dark gray, and light gray boxes, respectively) and follows the acetylcholinesterase homology (ACHE-like) domain (adapted from Di Jeso and Arvan) (28). NIS: one identified variant (arrows) in 1 of 13 different transmembrane domains (adapted from Kosugi et al.) (29). DUOX2: the different protein domains are as follows: the peroxidase-like domain is in the N-terminal region and the black boxes represent the transmembrane domains, the dark-gray boxes the EF-hand motifs, the light gray boxes the FAD-binding domain, the narrow white boxes the NADPH-binding domains, and the oval box the heme-binding site (adapted from Moreno et al.) (30). Mutations are shown by arrows in the various protein regions. DUOXA2: light gray boxes represent transmembrane domains (adapted from Grasberger and Refetoff) (31) and mutations are shown by arrows. TPO: the heme oxidase domain is shown in light gray with an oval representing its catalytic site (encoded by exons 8–10) and the transmembrane domain is represented in dark gray at the C-terminal end of the protein (adapted from Deladoey et al.) (32). The two *TPO* mutations are shown by arrows in the heme oxidase domain. PAX8: paired domain (dark gray box), residual paired type homeodomain (light gray box), and the repressor and activating domains defined by dark lines (adapted from Poleev et al.) (33). The three PAX8 variants are shown by arrows.

TGNS allowed the molecular diagnosis in the majority of patients (10/19) screened, providing a diagnostic yield of 53% **(** **Table 4**, **Figures 1**–**3** **)**. We identified of 14 novel variants on 24. Twelve/24 variants were causative in function of the context (genetic model, specificity of variants). *TG* was the most common site of mutations, followed by *DUOXA2* for causative variants (**Figure 1**). **Figure 2** shows the familial pedigrees and **Figure 3** the location of DH-causing mutations.

**Table 4:** Molecular and protein descriptions regarding variants found in patients with CH due to DH.

Patient	Gene	cDNA change	Amino acid change	Exon	Homozygous (ho)/Heterozygous (he)	Variant type	Protein Domain	Variant name - ExAC frequence/dbSNP or MAF and allele frequency in gnomAD	*In silico* prediction				Inheritance	Reference/ho or het/Functional study	Causative
									SIFT	Polyphen-2	Mutation Taster	CADD score			
1	TG	c.638+1 G>A		1_ex.5	He	splice donor region	Type 1 repeat	8_133885467_G_A- 0	–	–	–	25.9	carrier mother	Alzahrani et al. (16) / ho / no	Yes
	TG	c.886 C>T	p.R277X	7	He	premature stop codon	Type 1 repeat	rs121912648 - 0.0003625 - gnomAD 0.0003535	–	–	–	37	carrier father	Van de graaf et al. (17) / ho / no	Yes
2	TG	c.6701 C>A	p.A2215D	38	Ho	missense	ACHE-like domain	rs370991693 - 0.00004141 - gnomAD 0.00004245	Deleterious	Damaging	Disease causing	32	NA	Caputo et al. (18), Pardo et al. 2009 (19)/ het compound and ho/ deficient TG secretion, retention in cells	Yes
	DUOX2	c.601_602insG	p.G202Tfs99	6	He	frameshift	N-terminal peroxidase-like domain	rs565500345 - 0.001 - gnomAD 0.00009553	–	–	–	-1	NA	Pfarr et al. (20)-het compound/ No	No
3	TG	c.2132_2133insG	p.A693Gfs24	9	He	frameshift	Type 1 repeat	8_133899750_A_AG- 0	–	–	–	-1		No	Yes
	TG	c.4588 C>T	p.R1511X	22	He	premature stop	Type 1 repeat	rs121912646 - 0.00006591 -gnomAD 0.00009195	–	–	–	38	carrier father	Targovnik et al. (21), ho/ No	Yes
	SLC26A4	c.199 A>C	p.T67P	3	He	missense	–	7_107303775_A_C - 0	Deleterious	Benign	Polymorphism	8.8		No	No
	DUOX2	c.598 G>A	p.G200R	6	He	missense	N-terminal peroxidaselike domain	rs2467827 - 0.003 -gnomAD 0.0009541	Tolerated	Possibly damaging	Disease causing	11.2		No	No
4	DUOX2	c.3155 G>A	p.C1052Y	24		missense	–	rs76343591 - 0.004218 -gnomAD 0.001294	Deleterious	Possibly damaging	Polymorphism	24.7	NA	Tonacchera et al. (22)/ het compound / partial defect in H2O2 production	Yes
	TG	c.5299_5301 del.GAT	p.D1748del	27	He	no frameshift	Type 3 repeat	rs112749206 - 0.011 -gnomAD 0.004399	–	–		–	NA	Brust et al. (23)/ het compound / No	No
	TG	c.5370 A>G	p.I1771M	27	He	missense	Type 3 repeat	rs73710715 - 0.011 -gnomAD 0.004414	Benign	Tolerated	Polymorphism	0	NA	No	No
5	TG	c.648_649insG	p.A198Gfs14	6	He	frameshift	Type 1 repeat	8_133894118_T_TG - 0	–	–	–	-1	carrier mother	No	Yes
	TG	c.4588 C>T	p.R1511X	22	He	premature stop codon	Type 1 repeat	rs121912646 - 0.00006591 -gnomAD 0.00009195	–	–	–	38	carrier father	Targovnik et al. (21)/ ho /No	Yes
6	TG	c.7859 G>A	p.G2601D	45	He	missense	ACHE-like domain	rs978923522 - 0	Tolerated	Benign	Polymorphism	9.9	NA	No	No
7	TG	c.886C>T	p.R277X	7	He	premature stop codon	Type 1 repeat	rs121912648 - 0.0003625 -gnomAD 0.0003535	–	–		37	carrier father	Van de graaf et al. (17)/ ho / No	Yes
	TG	c.5182T>C	p.C1709R	26	He	missense	Type 3 repeat	rs37600169 - 0.00002471	Deleterious	Damaging	Disease causing	25.2	carrier mother	No	Yes
8	DUOXA2	c.205+2T>C		2_ex2	He	essential splicing	II transmembrane domain	rs201506037 - 0.00038 -gnomAD 0.0004583	–	–		25.2	NA	No	Yes
	DUOXA2	c.463C>G	p.L155V	4	He	coding	extracellular domain	rs201808443 - 0.00040 -gnomAD 0.0004595	Deleterious	Damaging	Disease causing	24.7	NA	No	Yes
	TG	c.2233_2234insT	p.L727Afs*3	10	He	frameshift	Type 1 repeat	eva_exac_8_133900286_C_CT - 0	–	–	–	-1	NA	No	?
	TG	c.3452delT	p.V1132Afs*31	16	He	frameshift	Type 1 repeat	rs766130576 - 0.00003	–	–	–		NA	No	?
9	TPO	c.866 T>C	p.F289S	8	He	missense	Heme peroxidase	2_1480904_T_C - 0	Deleterious	Damaging	Disease causing	25.2		No	No
10	TPO	c.1768+1insGTCTGCCAG		1_ex10	He	splice donor region	Heme peroxidase	2_1491764_G_GGTCTGC CAG - 0	–	–		-1		No	No
11	PAX8	c.101 T>A	p. I34N	3	He	missense	Paired box protein	2_114004421_A_T_0.00008331	Deleterious	Damaging	Disease causing	28.2	carrier mother	Lanzerath et al. (24)/ thyroid hypoplasia / het compound / No	Yes
12	PAX8	c.397C>T	p.R133W	5	He	missense	Paired box protein	2_114000348_G_A - 0	Deleterious	Damaging	Disease causing	35	NA	Vincenzi et al. (25) / het / Yes but not deleterious on TG	No
13	PAX8	c.658 C>T	p.R220X	7	He	premature stop codon	–	2_113999247_G_A - 0	–	–		37	carrier mother	Fu et al. (26) / het hypoplasia / No	No
14	SLC5A5	c.1183 G>A	p.G395R	10	Ho	missense	X transmembrane domain	rs121909180 - 0.00006595 -gnomAD 0.00004597	Deleterious	Damaging	Disease causing	33	carrier mother, father NA	Kosugi et al. (27)/ ho / no iodide uptake	Yes
15	SLC5A5	c.1183 G>A	p.G395R	10	Ho	missense	X transmembrane domain	rs121909180 - 0.00006595 -gnomAD 0.00004597	Deleterious	Damaging	Disease causing	33	carrier father and mother	Kosugi et al. (27)/ ho / no iodide uptake	Yes

NA, Not available.

? No definitive molecular diagnosis.

For TG, the amino acid positions are numbered after subtracting the 19-amino acid signal peptide.

#### *TG* Variants

Seven novel variants have been identified by TNGS in our study cohort, located mainly in the type 1 repeat protein domain and the acetylcholinesterase (ACHE)-like domain.

Two siblings (patient #1 and his brother) were compound heterozygous for two previously described *TG* mutations: an insertion in a splice site (c.638+1 G>A) leading to exon 5 skipping and a missense mutation (c.886C>T, pR277X) producing a premature stop codon (16, 17). Familial segregation and Sanger sequencing confirmed that one variant was transmitted by the mother and the other one by the father (**Table 4**). Another known homozygous *TG* mutation (c.6701C>A, p.A2215D) in the ACHE-like domain, associated with deficient TG secretion due to TG retention within the endoplasmic reticulum (18, 19) was identified in patient #2. Unfortunately, no parental DNA was available. Patient #3 was compound heterozygous for two *TG* mutations including the previously described c.4588 C>T mutation (inherited from the father) that produces a premature stop codon (p.R1511X) with skipping of the exon 22 domain (34). Exon 22 skipping may result in protein structure alterations responsible for protein retention within the cell. This nonsense mutation occurs in a CpG dinucleotide sequence and may be caused by deamination of a methylated cytosine resulting in a thymine (21). The other *TG* mutation was a novel variant, c.2132_2133 insG, in exon 9, responsible for a frameshift in the Type-1 domain. This c.2132_2133 insG mutation explains therefore along with the p.R1511X, the clinical phenotype of the patient. Patient #5 also had two *TG* mutations. One (c.4588 C>T, p.R1511X) was a previously described mutation (34) for which no functional studies are available, inherited from the father, and responsible for a stop codon. The other, inherited from the mother, was a novel G insertion in exon 6 leading to a frameshift in the Type 1 TG domain and to a premature stop codon. The previously reported c.886 C>T mutation (p.R277X), also found in patient #1, was identified in patient #7 along with a novel missense mutation that replaces a cysteine with an arginine (c.5182 T>C, p.C1709R), located in the type 3 repeat domain of TG, and responsible for absence of a disulfide bond that may alter the conformation of TG. A novel *TG* monoallelic variant (c.7859G>A, p.G2601D) located in the ACHE-like domain was found in another patient (patient #6); its pathogenicity cannot be ascertained *in silico.*

#### *DUOX2* and *DUOXA2* Variants

TGNS identified one case (patient #4) with a probably causative biallelic *DUOX2* mutation leading to partial deficiency in H_2_O_2_ production (22). Interestingly this patient had also two heterozygous *TG* variants located in the same type-3 protein domain, of which one—a 3-bp (GAT) deletion at amino acid position 1,767—has been reported previously (23) and the other is novel (c.5370A>G, p.I1771M); suggesting that accumulation of pathogenic variants may lead to CH.

In our study cohort, patients #2 and #3 besides *TG* mutations, carried a monoallelic *DUOX2* variant not located in *DUOX2* functional domains or hot-spot mutations. The *DUOX2* variant of patient #2 has been previously described in patients with DH, without any functional study (20).

The accumulation of variants in different DH genes applies on other cases, as in patient #8 heterozygous for four novel variants in *TG* and *DUOXA2*. The two *TG* variants, c.2233_2234insT and c.3452delT, were in the type 1 repeat domain. One *DUOXA2* variant, c.205+2 T>C, was in an essential splice site. *In silico* predictions predictive algorithms show an abolishment of natural splice donor site, suggesting a splicing effect of the variation. The other *DUOXA2* mutation, c.463 C>G, p.L155V, was a missense variant in the larger extracellular domain. The patient had a GIS and a positive perchlorate discharge test. Although the two *DUOXA2* mutations may explain the organification defect, a pathogenic effect of the two *TG* mutations cannot be ruled out, particularly as no TG assay was performed at diagnosis.

#### *TPO* Variants

We did not identify any biallelic mutations but only two novel monoallelic variants. Monoallelic *TPO* mutations have been reported to cause DH with showed monoallelic expression (35). The first *TPO* variant c.866 T>C, p.F289S, is in exon 8, encoding for the catalytic domain (patient #9). *In silico* prediction tools suggested a deleterious or disease-causing effect. Monoallelic expression of the *TPO* mutation in patient #9 cannot be ruled out, as no thyroid tissue study was performed. Familial segregation for patient #9 showed that mother carried this *TPO* variant despite a normal thyroid function. The second variant (patient #10) is an insertion (c.1768+1insGTCTGCCAG), after exon 10. This variation does not affect the main splice donor site, but because of the duplicated sequence it creates a putative new splice donor site, which is the same as the previous site. Its pathogenicity remains questionable, as ideally functional data would be helpful.

#### *PAX8* Variants

*PAX8* mutations have been chiefly described in patients with CH and thyroid dysgenesis, some of whom also have renal and urinary malformations (36). However, mutations in the *PAX8* binding domain have been found in patients with CH and DH characterized by defective iodide organification and positive perchlorate discharge test or partial iodide transport defect, with normal-sized thyroid gland (37, 38). Three previously reported variants have been identified in three cases of cohort; two of them in the DNA binding domain. One case (patient #11), with GIS and a positive perchlorate discharge test, was heterozygous for a mutation located in the binding domain of *PAX8* (c.101T>A, p.I34N). This mutation has been reported previously in a patient with CH and thyroid gland hypoplasia (24), who was also heterozygous for another *PAX8* mutation (p.V35I), in the DNA binding domain. Although no functional data are available, given its location in the binding domain, the p.I34N mutation probably impairs transactivation of *TG* or *TPO*. Moreover, this variant was inherited from the mother, who also had CH and GIS. Patient #12 was heterozygous for a missense variant (c.397C>T, p.R133W) at the end of the *PAX8* DNA binding domain. This previously described mutation had no deleterious effect on TG transactivation or with the synergism between PAX8 and NKX2-1 (25). Pathogenicity is unclear, for the third variant (patient #13) (c.658C>T, p.R220X) located after the *PAX8* binding domain and responsible for a premature stop codon; described previously in a single patient who did not undergo functional studies and whose phenotype is unclear (26). In addition, the mutation was inherited from the mother, who has normal thyroid function, position, and size.

#### *SLC5A5/NIS* Variants

The two Tunisian patients (#14 and #15), born to consanguineous parents, were homozygous for the same *SLC5A5* mutation, c.1183 G>A, p.G395R. In a previous study of this homozygous mutation, no perchlorate-sensitive iodide uptake was observed in COS-7 cells transfected with the mutant G395R NIS cDNA (27). Moreover, Dohan et al. have analyzed the effect of the p.G395R mutation allowing valuable insights into the structure-function and mechanistic properties of NIS (39). Amino acid substitutions at position 395 showed that the presence of an uncharged amino acid residue with a small side chain at position 395 is required for NIS function, suggesting that glycine 395 is located in a tightly packed region of NIS. For family of patient #14, the mother carries the variant; father’s DNA was not available. The brother diagnosed also with CH at 8 months of age is homozygous for the mutation. For patient #15, both parents are heterozygous as well as the healthy sibling.

#### Unsolved Cases With “No Causative” Variants

In four patients (#16–19), our filter prioritization strategy identified no mutations. Among these patients, three had goiter at diagnosis and two had familial CH, including one born to consanguineous parents.

### Literature Review

We reviewed the literature by searching PubMed with the following terms: “hypothyroidism AND mutations”, “hypothyroidism AND mutations AND sequencing”, and “hypothyroidism AND dyshormonogenesis”. We excluded articles that did not provide NM accession numbers, detailed clinical data, and/or a detailed genetic analysis. Nearly 400 patients with hypothyroidism and GIS, with or without goiter, have been evaluated using either TNGS including a mean of 14 genes or whole exome sequencing (7, 8, 10–13, 40, 41). Most patients underwent thyroid sonography to assess gland position and size. Selected variants were missense mutations and nonsense mutations in coding regions and splice sites. Variants in untranslated regions or noncoding RNA and synonymous variants were disregarded. The filters used were based on minor allele frequency [mainly MAF of 0.01 or 0.02, and 0.001 in only one study (41)] and *in silico* prediction tool results (usually SIFT and Polyphen-2 and less often Mutation Taster, Mutation Assessor, FATHMM, GERP score, CONDEL, and PROVEAN). Variants were classified based on frequency, *in silico* prediction tool results, pedigree segregation, and functional studies, as available. In one study, a score from A to C was used to stratify variants according to these criteria (8). The frequency of patients with mutations in known genes varied from 20 to 60% in cohorts including patients of various ethnicities (Korean, Chinese, Finnish, Italian, Saudi Arabian, Russian, and multiethnic). **Table 5** recapitulates the main clinical and molecular NGS studies published so far.

**Table 5:** Literature review.

	Park, Ann Lab Med 2016	Lof, Thyroid, 2016	Jiang, Eur J Med Genet, 2016	Nicholas, J Clin Endocrinol Metab, 2016	de Filippis, Hum Mol Genet, 2017	Zou, JCEM, 2018	Sun, EJE, 2018	Makretskaya, PlosOne, 2018
**Sequencing approach**	multiplexPCR	TNGS	TNGS	TNGS, WES	TNGS	WES	TNGS	TNGS and MLPA
**genes**	*TPO, TSHR, DUOX2, DUOXA2*, *NIS, PAX8*	*TG, TPO, TSHR, DUOX2, IYD, NIS, PDS, DUOX1*, *NKX2-5, PAX8, TRH, TRHR, TSHB*	*TG, TPO, TSHR, DUOX2, IYD, NIS, NKX2-5, PAX8, NKX2-1, FOXE1, SLC26A4, GNAS*	*TG, TPO, TSHR, DUOX2, DUOXA2, IYD, NIS, PDS*	*TG, TPO, TSHR, DUOX2, DUOXA2, PDS, PAX8, NKX2-1, FOXE1, GLIS3, JAG1*	*TG, TPO, TSHR, DUOX2, PDS, PAX8, NKX2-1, SLC26A7, TSHB, CDCA8, HOXB3 (mutated genes)*	*TG, TPO, TSHR, DUOX2, DUOXA2, IYD, NIS, PDS, PAX8, NKX2-1, FOXE1, DIO1*,	*TG, TPO, TSHR, DUOX2, DUOXA2, IYD, NIS, PDS, PAX8, NKX2-1, FOXE1, NKX2-5*
causative genes for DH or DT	6	13	12	8	11	WES	DIO2, DUOX1, DUOXA1, THRB, THRA, GNAS, SLC16A2, HHEX, NKX2-5	12
**Patients**								
patients with GIS	112 (CH newborn screening) + 58 CH (outpatients, adult follow-up)=170	26 (15 sporadic goiter, athyreosis, hypoplasia; 11 families GIS, athyreosis, hypoplasia)	12	49	94 (goiter, GIS)	30 DH (goiter, GIS)	110 CH (37 trios), 21 goiter, 51 GIS, 10 TD, 28 NA	243 CH
patients with TD					83	25 TD (including 1 CeH)		
clinical description DH/TD	CH, thyroid scan and ultrasound for outpatients without data	thyroid US if data available, TG levels	CH, newborn screening, ultrasonic scanning	CH, newborn screening, thyroid imaging (GIS or goiter)	CH, newborn screening, thyroid US and/or scintigraphy	CH, newborn screening, thyroid US and/or scintigraphy	CH, newborn screening	severe CH (TSH at diagnosis >90mU/L)
ethnicity	Korean	Finnish	Chinese	Multiethnic origin	Italian	Saudi Arabia	Chinese	Russian
**Filters used variants analysis**								
frequency	0.01	0.01	0.02	0.02	0.01		0.01	0.01
types of variants	missense, nonsense mutations in coding regions, without variants UTR, non coding RNA, without synonymous variants; deleterious/damaging	coding regions or splicing exons, unknown and variant <1%; variant classification with selection of variants with high and moderate effect on gene function	no intronic and synonymous, "tolerated" or "benign" variants were excluded; rare, deleterious, putative deleterious variants	variants affecting protein coding sequence/splicing, possibly damaging and above	nonsense, frameshift, splice site, missense; deleterious in 5 out of 7 algorithms of the dbSNP database	VUS: mutations if <0.01, damaging or disease causing in 3/4 prediction tools, strict segregation, biallelic: disease causing	functional variants (altering protein), without UTR or intergenic variants, synonymous	Benign and "likely benign" variants were excluded
*in silico* prediction	SIFT, PolyPhen-2, Mutation Taster, Mutation Assesor, FATHMM, GERP score >2, HGMD	Condel	SIFT, PolyPhen-2, CONDEL	Ensembl VEP, SIFT, PolyPhen-2, GERP	SIFT, Polyphen2, Mutation Taster, Mutation Assessor, LRT, FATHMM, NetGen2v2.4 ESE Finder 2.0 for intron variants	Mutation Taster, PolyPhen-2, SIFT, PROVEAN	Function using ANNOVAR (UCSC)	ACMG guidelines
pedigree segregation studies	for unknown mutations	in familial cases	no	when possible	in families	yes	when possible	no
functional testing	no	protein modeling and *in vitro* experiments	no	when possible, protein modeling	if published	no	no	no
**Conclusions**								
% diagnosed patients according to authors criteria	31% (53/170), mono and biallelic variants	54.5% (6/11 familial cases), 20% (3/15 sporadic cases) with accurately analysis by variant	91.7 % (11/12 patients), mono and biallelic variants	59% solved cases, (29/49), 22% ambiguous cases (11/49), 18% unsolved cases (9/49) with accurate analysis by variant	103/177 patients with a rare variant, 5 cases with monogenic recessive forms, 39 patients with oligogenic model	60% (18/30) in DH (biallelic mutations), 37.5% (9/24) for TD, if *HOXB3* causative: 40% (10/25) for TD	51.8% (57/110 patients) with biallelic mutations, recessive manner of inheritance	37.9% (92/243) with variants considered as pathogenic/likely pathogenic including VUS mono-and biallelic
final conclusions of article	*DUOX2:* frequent cause of CH in the Korean population	TNGS: cost-effective, efficient, and multigenic screening, classification of variants (based on segregation, literature and *in vitro* experiments)	high prevalence of *DUOX2* mutations (83.3%) in central China, all patients were biallelic, tri-allelic or compound mutations in other genes	biallelic variants *TPO/TG*, *PDS/TPO*, *DUOX2/TG*, *TG+TPO :* more severe CH phenotype, triallelic variant are frequent	Oligogenic models of CH with causative genes, CH population is significantly enriched with rare/low frequency alleles in the 11 CH- related genes	TG mutations: more frequent in DH with diagnostic yield of 66% in mutated DH patients (12/18) and 40% for all patients (12/30), *TSHR* more frequent in TD (60% of TD mutated patients 6/10 and 25% for 6/24 patients), SLC26A7 : new candidate gene for DH ?	Diagnostic yield of 51,82% (for biallelic mutations), high frequency of *DUOX2* mutations compared to Caucasian population	Majority of variants in DH genes responsible for severe CH

CeH, Central hypothyroidism.

VUS, variant of incertain signifiance.

ACMG, American College of Medical Genetics and Genomics.

## Discussion

We used TNGS to perform comprehensive genetic screening of a well-characterized cohort of patients with CH due to DH, including patients with GIS and/or goiter at diagnosis and/or a positive perchlorate discharge test. The proportion of patients with identified disease-causing mutations was 53%. *TG* mutations predominated, as described previously (7, 13). We found 25% of *DUOX2* and *DUOXA2* variants as in others European countries (42). *DUOX2* mutations were uncommon in our cohort, in contrast to studies in Asians showing a prevalence of 60% (12). This could be explained by the absence of patients of Asian ethnicity in our cohort and the detailed phenotypic and molecular description and assessment of variants. The pathogenicity of each variant was carefully evaluated based on clinical data including correlations with clinical phenotypes, previously published information, availability of functional studies, *in silico* prediction tool results, and location of variants in regions of interest of the protein. Interestingly we detected no causative *DEHAL1/IYD* or *SLC26A4/PDS* variants, suggesting that these may be rarely responsible for CH due to DH, depending usually on iodine uptake or associated with syndromic features as in the case of Pendred syndrome (4, 6). The proportion of patients with identified mutations differed between familial and sporadic cases (62 versus 54%, respectively).

The biallelic *TG* mutations identified in patient #2 have been reported previously and were consistent with the clinical presentation of goiter, low thyroglobulin levels, elevated iodide uptake, and normal perchlorate discharge test. Similarly, in patients #1 and #3, the goiter in utero or GIS with normal radionuclide uptake and nearly normal perchlorate discharge test are consistent with a *TG* mutation. Interestingly, the perchlorate discharge test result in patient #5 was elevated. Variable perchlorate discharge test values and partial iodide organification defects have been reported in patients with *TG* mutations (43, 44). When available, thyroglobulin values were in agreement with the molecular diagnosis, as observed in patients #2 and #7. This finding confirms that very low thyroglobulin levels are a good indication for *TG* mutation screening (45).

Biallelic *DUOX2* mutations explaining the DH were detected in a single patient (#4), who had a goiter and a positive perchlorate discharge test. Monoallelic *DUOX2* variants were identified combined with biallelic mutations in *TG* gene. The pathogenic role of these variants and their contribution, if any, to disease severity is difficult to determine. We identified two heterozygous *TPO* variants (patients #9 and #10) as the only disease-causing candidates. DH due to monoallelic *TPO* variants has been reported (35). Monoallelic expression in thyroid tissue and/or other, unidentified genetic factors may explain the phenotype. Indeed, the *TPO* variant of the patient #9 (c.866T>C, considered as deleterious through three in silico predictive algorithms) could be disease-causing if associated with another *TPO* variant or if there is monoallelic expression. However, given the uncertainty regarding the molecular diagnosis and the absence of functional data, the contribution to the CH phenotype of patients remains unclear.

We detected compound heterozygosity for two different novel *DUOXA2* variants in a patient with DH and a partial iodide organification defect (patient #8). *DUOXA2* mutations are a rare cause of DH, and only seven variants have been reported so far (5). Interestingly, we identified three different *PAX8* variants in three patients (#11, #12, and #13), including two variants located in the DNA-binding domain. A single variant (p.I34N) was considered causative, based on location and familial segregation. However, p.R133W is also located in the binding domain, although a previously reported functional study found no evidence of a causative effect (25). The variant p.I34N in *PAX8* of patient #11 was causative probably due to its involvement in transactivation of DUOX2 and TPO leading to defect of iodide organification. *PAX8* mutations cause thyroid dysgenesis and some mutations were compatible with dyshormonogenesis as already described (37, 38).

Our literature review of studies of the molecular diagnosis of CH using NGS techniques including whole exome sequencing showed that differences in mutation frequencies across cohorts were chiefly ascribable to differences in ethnicity. Korean, Chinese, Finnish, Italian, Russian, or Saudi Arabian patients were studied. A single study included patients of different ethnicities (7). In two studies that distinguished between familial and sporadic cases, mutations were identified in about 54% of familial cases and 20–22% of sporadic cases (7, 8). No such difference was observed in our cohort. Furthermore, frequency differences across cohorts depend on the type of variant classification. Some studies determined the number of variants without differentiating variants with recessive versus dominant inheritance. Inheritance is usually recessive for variants responsible for CH with DH (11, 12, 40). The frequency of pathogenic variants was therefore overestimated. When identifying genetic causes of CH, the challenge consists in using appropriate criteria to select variants for TNGS screening. Interestingly, the diagnostic yield of TNGS in our study was 53%, in keeping with the results of previous studies that used a similar filter prioritization strategy (**Table 5**). Reports of digenic variants in several genes (*TPO/TG, PDS/TPO, DUOX2/TG*) suggest a pathogenic effect of variant accumulation, with the occurrence of DH. Oligogenic models involving CH-causing genes have been developed (10). We also found two or three mutated genes in several patients, although presence of a single recessive mutation was sufficient to cause DH, without the presence of the other monoallelic variants.

Combining scintigraphy and thyroid ultrasound in the individual patient improves diagnostic accuracy and guides molecular studies. In our cohort, positive TNGS findings correlated well with thyroid radionuclide uptake and perchlorate discharge test results. TNGS after newborn screening and CH confirmation may be a valid strategy for rapidly obtaining the accurate diagnosis of CH due to DH. TNGS, if available, may serve as a diagnostic alternative to thyroid scintigraphy, which is a time-consuming and invasive method. However, this approach should not delay treatment initiation and appropriate clinical care of patients.

In conclusion, in a well-characterized cohort of patients with DH, our TNGS approach provided the molecular diagnosis and shed light on genetic cause in 53% of cases. Several novel mutations were detected, half of which were causative for DH. Our analysis of the identified variants was based on both a detailed phenotypic description and an in-depth assessment of causality. TNGS is a rapid and cost-effective method for screening patients with CH. Patients whose TNGS results fail to provide the molecular diagnosis can then be assessed using other NGS approaches, i.e., whole exome sequencing or whole genome sequencing, with the goal of identifying new candidate genes.

## Data Availability Statement

The original contributions presented in the study are publicly available. This data can be found here: Clinical data: ClinVar accession numbers: VCV000888352, VCV000712030, VCV000631732, VCV000372358, VCV000361974, VCV000279800, VCV000265105, VCV000012706, VCV000012695, VCV000012691, VCV000007670.

## Ethics Statement

The studies involving human participants were reviewed and approved by French Biomedecine Agency. Written informed consent to participate in this study was provided by the participants’ legal guardian/next of kin.

## Author Contributions

AC and MP coordinated and instigated the study. AS, CT, GP, MM, SB, MD, RR, PB, MH, and NB provided clinical samples and data. AS and GAHC performed molecular studies. AC, AS, and GAHC analyzed the data. CB-F and SH coordinated NGS procedure. PN and CF gave bioinformatics support. AC, AS, and MP draft and finalized the manuscript with the help of GS and DK. All authors contributed to the article and approved the submitted version.

## Funding

AS was supported in part by an Onassis Foundation Grant and a European Society for Pediatric Endocrinology (ESPE) Research Fellowship grant. AC and MP received financial support from three corporations (EDF, Sandoz SAS, and Merck Serono France) and from the non-profit Princess Grace Foundation of Monaco. GS was supported by ESPE. Funders had no role in study design, data collection and analysis, decision to publish, or preparation of the manuscript.

## Conflict of Interest

The authors declare that the research was conducted in the absence of any commercial or financial relationships that could be construed as a potential conflict of interest.

## Abbreviations

CH, congenital hypothyroidism; DH, dyshormonogenesis; GIS, gland *in situ*; MAF, minor allele frequency; NGS, Next generation Sequencing.

## References

[1] Barry Y Bonaldi C Goulet V Coutant R Léger J Paty AC. Increased incidence of congenital hypothyroidism in France from 1982 to 2012: a nationwide multicenter analysis. Ann Epidemiol (2016) 26:100–5. 10.1016/j.annepidem.2015.11.005 26775052

[2] Deladoëy J Ruel J Giguère Y Van Vliet G. Is the incidence of congenital hypothyroidism really increasing? A 20-year retrospective population-based study in Québec. J Clin Endocrinol Metab (2011) 96:2422–9. 10.1210/jc.2011-1073 21632812

[3] Stoupa A Kariyawasam D Carré A Polak M. Update of Thyroid Developmental Genes. Endocrinol Metab Clin North Am (2016) 45:243–54. 10.1016/j.ecl.2016.01.007 27241962

[4] Targovnik HM Citterio CE Rivolta CM. Iodide handling disorders (NIS, TPO, TG, IYD). Best Pract Res Clin Endocrinol Metab (2017) 31:195–212. 10.1016/j.beem.2017.03.006 28648508

[5] Muzza M Fugazzola L. Disorders of H2O2 generation. Best Pract Res Clin Endocrinol Metab (2017) 31:225–40. 10.1016/j.beem.2017.04.006 28648510

[6] Wémeau JL Kopp P. Pendred syndrome. Best Pract Res Clin Endocrinol Metab (2017) 31:213–24. 10.1016/j.beem.2017.04.011 28648509

[7] Nicholas AK Serra EG Cangul H Alyaarubi S Ullah I Schoenmakers E. Comprehensive Screening of Eight Known Causative Genes in Congenital Hypothyroidism With Gland-in-Situ. J Clin Endocrinol Metab (2016) 101:4521–31. 10.1210/jc.2016-1879 PMC5155683 27525530

[8] Löf C Patyra K Kuulasmaa T Vangipurapu J Undeutsch H Jaeschke H. Detection of Novel Gene Variants Associated with Congenital Hypothyroidism in a Finnish Patient Cohort. Thyroid (2016) 26:1215–24. 10.1089/thy.2016.0016 PMC5036323 27373559

[9] Fan X Fu C Shen Y Li C Luo S Li Q. Next-generation sequencing analysis of twelve known causative genes in congenital hypothyroidism. Clin Chim Acta (2016) 468:76–80. 10.1016/j.cca.2017.02.009 28215547

[10] de Filippis T Gelmini G Paraboschi E Vigone MC Di Frenna M Marelli F. A frequent oligogenic involvement in congenital hypothyroidism. Hum Mol Genet (2017) 26:2507–14. 10.1093/hmg/ddx145 28444304

[11] Park KJ Park HK Kim YJ Lee KR Park JH Park JH. DUOX2 Mutations Are Frequently Associated With Congenital Hypothyroidism in the Korean Population. Ann Lab Med (2016) 36:145–53. 10.3343/alm.2016.36.2.145 PMC4713848 26709262

[12] Sun F Zhang JX Yang CY Gao GQ Zhu WB Han B. The genetic characteristics of congenital hypothyroidism in China by comprehensive screening of 21 candidate genes. Eur J Endocrinol (2018) 178:623–33. 10.1530/EJE-17-1017 PMC5958289 29650690

[13] Zou M Alzahrani AS Al-Odaib A Alqahtani MA Babiker O Al-Rijjal RA. Molecular Analysis of Congenital Hypothyroidism in Saudi Arabia: SLC26A7 Mutation Is a Novel Defect in Thyroid Dyshormonogenesis. J Clin Endocrinol Metab (2018) 103:1889–98. 10.1210/jc.2017-02202 29546359

[14] Stoupa A Adam F Kariyawasam D Strassel C Gawade S Szinnai G. TUBB1 mutations cause thyroid dysgenesis associated with abnormal platelet physiology. EMBO Mol Med (2018) 10:e9569. 10.15252/emmm.201809569 30446499 PMC6284387

[15] Leger J Olivieri A Donaldson M Torresani T Krude H van Vliet G. European Society for Paediatric Endocrinology consensus guidelines on screening, diagnosis, and management of congenital hypothyroidism. J Clin Endocrinol Metab (2014) 99:363–84. 10.1210/jc.2013-1891 PMC4207909 24446653

[16] Alzahrani AS Baitei EY Zou M Shi Y. Clinical case seminar: metastatic follicular thyroid carcinoma arising from congenital goiter as a result of a novel splice donor site mutation in the thyroglobulin gene. J Clin Endocrinol Metab (2006) 91:740–6. 10.1210/jc.2005-2302 16403815

[17] van de Graaf SA Ris-Stalpers C Veenboer GJ Cammenga M Santos C Targovnik HM. A premature stopcodon in thyroglobulin messenger RNA results in familial goiter and moderate hypothyroidism. J Clin Endocrinol Metab (1999) 84:2537–42. 10.1210/jcem.84.7.5862 10404833

[18] Caputo M Rivolta CM Esperante SA Gruñeiro-Papendieck L Chiesa A Pellizas CG. Congenital hypothyroidism with goitre caused by new mutations in the thyroglobulin gene. Clin Endocrinol (Oxf) (2007) 67:351–7. 10.1111/j.1365-2265.2007.02889.x 17532758

[19] Pardo V Vono-Toniolo J Rubio IG Knobel M Possato RF Targovnik HM. The p.A2215D thyroglobulin gene mutation leads to deficient synthesis and secretion of the mutated protein and congenital hypothyroidism with wide phenotype variation. J Clin Endocrinol Metab (2009) 94:2938–44. 10.1210/jc.2009-0150 19509106

[20] Pfarr N Korsch E Kaspers S Herbst A Stach A Zimmer C. Congenital hypothyroidism caused by new mutations in the thyroid oxidase 2 (THOX2) gene. Clin Endocrinol (Oxf) (2006) 65:810–5. 10.1111/j.1365-2265.2006.02672.x 17121535

[21] Targovnik HM Esperante SA Rivolta CM. Genetics and phenomics of hypothyroidism and goiter due to thyroglobulin mutations. Mol Cell Endocrinol (2010) 322:44–55. 10.1016/j.mce.2010.01.009 20093166

[22] Tonacchera M De Marco G Agretti P Montanelli L Di Cosmo C Freitas Ferreira AC. Identification and functional studies of two new dual-oxidase 2 (DUOX2) mutations in a child with congenital hypothyroidism and a eutopic normal-size thyroid gland. J Clin Endocrinol Metab (2009) 94:4309–14. 10.1210/jc.2009-0426 19789206

[23] Brust ES Beltrao CB Watanabe T. New heterozygous mutations in thyroglobulin gene in patients with congenital hypothyroidism. Endoc Rev (2011). The endocrine society’s 93rd annual meeting.

[24] Lanzerath K Bettendorf M Haag C Kneppo C Schulze E Grulich-Henn J. Screening for Pax8 mutations in patients with congenital hypothyroidism in South-West Germany. Horm Res (2006) 66:96–100. 10.1159/000093799 16763387

[25] Vincenzi M Camilot M Ferrarini E Teofoli F Venturi G Gaudino R. Identification of a novel pax8 gene sequence variant in four members of the same family: from congenital hypothyroidism with thyroid hypoplasia to mild subclinical hypothyroidism. BMC Endocr Disord (2014) 14. 10.1186/1472-6823-14-69 PMC4142740 25146893

[26] Fu C Chen R Zhang S Luo S Wang J Chen Y. PAX8 pathogenic variants in Chinese patients with congenital hypothyroidism. Clin Chim Acta (2015) 450:322–6. 10.1016/j.cca.2015.09.008 26362610

[27] Kosugi S Bhayana S Dean HJ. A novel mutation in the sodium/iodide symporter gene in the largest family with iodide transport defect. J Clin Endocrinol Metab (1999) 84:3248–53. 10.1210/jcem.84.9.5971 10487695

[28] Di Jeso B Arvan P. Thyroglobulin From Molecular and Cellular Biology to Clinical Endocrinology. Endocr Rev (2016) 37:2–36. 10.1210/er.2015-1090 26595189 PMC4740344

[29] Kosugi S Okamoto H Tamada A Sanchez-franco F. A Novel Peculiar Mutation in the Sodium/Iodide Symporter Gene in Spanish Siblings with Iodide Transport Defect. J Clin Endocrinol Metab (2002) 87:3830–6. 10.1210/jcem.87.8.8767 12161518

[30] Moreno J Bikker H Kempers M van Trotsenburg P Baas F de Vijlder J. Inactivating mutations in the gene for thyroid oxidase 2 (thox2) and congenital hypothyroidism. N Engl J Med (2002) 347:95–102. 10.1056/NEJMoa012752 12110737

[31] Grasberger H Refetoff S. Identification of the maturation factor for dual oxidase. J Biol Chem (2006) 281:18269–72. 10.1074/jbc.C600095200 16651268

[32] Deladoëy J Pfarr N Vuissoz JM Parma J Vassart G Biesterfeld S. Pseudodominant Inheritance of Goitrous Congenital Hypothyroidism Caused by TPO Mutations: Molecular and in Silico Studies. J Clin Endocrinol Metab (2008) 93:627–33. 10.1210/jc.2007-2276 18029453

[33] Poleev A Okladnova O Musti AM Schneider S Royer-Pokora B Plachov D. Determination of functional domains of the human transcription factor PAX8 responsible for its nuclear localization and transactivating potential. Eur J Biochem (1997) 247:860–9. 10.1111/j.1432-1033.1997.00860.x 9288908

[34] Targovnik HM Medeiros-Neto G Varela V Cochaux P Wajchenberg BL Vassart G. A nonsense mutation causes human hereditary congenital goiter with preferential production of a 171-nucleotide-deleted thyroglobulin ribonucleic acid messenger. J Clin Endocrinol Metab (1993) 77:210–5. 10.1210/jcem.77.1.8325944 8325944

[35] Fugazzola L Cerutti N Mannavola D Vannucchi G Fallini C Persani L. Monoallelic expression of mutant thyroid peroxidase allele causing total iodide organification defect. J Clin Endocrinol Metab (2003) 88:3264–71. 10.1210/jc.2002-021377 12843174

[36] Ramos HE Carré A Chevrier L Szinnai G Tron E Cerqueira TL. Extreme phenotypic variability of thyroid dysgenesis in six new cases of congenital hypothyroidism due to PAX8 gene loss-of-function mutations. Eur J Endocrinol (2014) 171:499–507. 10.1530/EJE-13-1006 25214233

[37] Jo W Ishizu K Fujieda K Tajima T. Congenital Hypothyroidism Caused by a PAX8 Gene Mutation Manifested as Sodium/Iodide Symporter Gene Defect. J Thyroid Res (2010) 2010:619013. 10.4061/2010/619013 21048839 PMC2956980

[38] Meeus L Gilbert B Rydlewski C Parma J Roussie AL Abramowicz M. Characterization of a novel loss of function mutation of PAX8 in a familial case of congenital hypothyroidism with in-place, normal-sized thyroid. J Clin Endocrinol Metab (2004) 89:4285–91. 10.1210/jc.2004-0166 15356023

[39] Dohán O Gavrielides MV Ginter C Amzel LM Carrasco N. Na(+)/I(-) symporter activity requires a small and uncharged amino acid residue at position 395. Mol Endocrinol (2002) 16:1893–902. 10.1210/me.2002-0071 12145342

[40] Jiang H Wu J Ke S Hu Y Fei A Zhen Y. High prevalence of DUOX2 gene mutations among children with congenital hypothyroidism in central China. Eur J Med Genet (2016) 59:526–31. 10.1016/j.ejmg.2016.07.004 27498126

[41] Makretskaya N Bezlepkina O Kolodkina A Kiyaev A Vasilyev EV Petrov V. High frequency of mutations in “dyshormonogenesis genes” in severe congenital hypothyroidism. PloS One (2018) 13:e0204323. 10.1371/journal.pone.0204323 30240412 PMC6150524

[42] Peters C Nicholas AK Schoenmakers E Lyons G Langham S Serra EG. DUOX2/DUOXA2 Mutations Frequently Cause Congenital Hypothyroidism that Evades Detection on Newborn Screening in the United Kingdom. Thyroid (2019) 29:790–801. 10.1089/thy.2018.0587 31044655 PMC6588112

[43] Niepomniszcze H Medeiros-Neto GA Refetoff S Degroot LJ Fang VS. Familial goitre with partial iodine organification defect, lack of thyroglobulin, and high levels of thyroid peroxidase. Clin Endocrinol (Oxf) (1977) 6:27–39. 10.1111/j.1365-2265.1977.tb01993.x 844215

[44] Niu DM Hsu JH Chong KW Huang CH Lu YH Kao CH. Six new mutations of the thyroglobulin gene discovered in taiwanese children presenting with thyroid dyshormonogenesis. J Clin Endocrinol Metab (2009) 94:5045–52. 10.1210/jc.2009-0646 19837936

[45] Targovnik HM Citterio CE Rivolta CM. Thyroglobulin gene mutations in congenital hypothyroidism. Horm Res Paediatr (2011) 75:311–21. 10.1159/000324882 21372558

